# The Coordination Abilities of Three Novel Analogues of Saliva Peptides: The Influence of Structural Modification on the Copper Binding

**DOI:** 10.1007/s10989-016-9569-x

**Published:** 2017-02-04

**Authors:** Aleksandra Kotynia, Edward Krzyżak, Elżbieta Kamysz, Małgorzata Sobocińska, Justyna Brasuń

**Affiliations:** 1 Department of Inorganic Chemistry, Wroclaw Medical University, Borowska 211a, 50-552 Wroclaw, Poland; 2 Laboratory of Chemistry of Biological Macromolecules, Department of Molecular Biotechnology, Faculty of Chemistry, University of Gdansk, Wita Stwosza 63, 80-308 Gdańsk, Poland

**Keywords:** Analogues of salivary pentapeptides, Stability constants of copper(II) complexes, Peptide synthesis, UV–Vis and CD spectroscopy

## Abstract

Three novel analogues of salivary peptides as sialorphin (QHNPR) and opiorphin (QRFSR) were synthesized by the solid-phase method. The sequences of these ligands were following: AHNPR, QANPR and QRFPR. The aim of our work was investigation in what way some structural modifications may impact on coordination abilities of studied peptides. In this work we presented the interaction of pentapeptides with copper(II) ions in wide range of pH. To determine the coordination model of ligands there were carried out several studies by spectroscopy (UV–Vis, CD) methods and potentiometric measurements.

## Introduction

Human opiorphin (QRFSR) and rat sialorphin (QHNPR) are natural inhibitors of enkephalin catabolism. Opiorphin was identified in human saliva and it showed beneficial effects in pain management, antidepressant-like actions as well as in involvement in colonic motility and erectile physiology (Davies 2009; Thanawala et al. 2008; Tian et al. 2009; Tong et al. 2008; Wisner et al. 2006; Yang et al. 2011). Sialorphin is a peptide synthesized predominantly in the submandibular gland and prostate of adult rats in response to androgen steroids, and is released locally and systemically in response to stress (Rougeot et al. 2003). Sialorphin exhibits a potent analgesic activity and plays a crucial role in the control of social behavior, pain perception and in sexual behavior (Davies et al. 2007; Messaoudi et al. 2004; Rougeot et al. 2003).

The metal ions such as Cu(II), Ni(II), and Zn(II) are essential and beneficial elements in mammalian nutrition, are required in a number of enzymatic reactions and can be important regulators of activity of many peptides (Barnea 1989; Cummins and O’Connor 1998; Gerega et al. 1988).

Copper ions play an important role in the progression of neurodegenerative diseases (Migliorini et al. 2014; Valensin et al. 2016), affect the development and progression of different cancers (Khanna et al. 2013), exhibit anti-inflammatory effects (Lewis 1984), are essential for bone integrity and elasticity (Sierpinska et al. 2014), influence tooth demineralization and remineralization (Abdullah et al. 2006; Brookes et al. 2003) as well as aroma perception by affecting volatility of aroma compounds in the mouth through interaction with salivary components, especially proteins (Hong et al. 2006). Human body is provided in various complicated mechanisms involving interactions with proteins and other molecules that make copper available during its deficiency, as well as enable elimination of excessive copper through absorption, transport, distribution, storage and excretion. Any failure of the copper homeostasis results inevitably in severe diseases. Elevated levels of salivary copper are observed in patients with oral submucous fibrosis, oral leukoplakia and oral cancer (Ayinampudi and Narsimhan 2012; Trivedy et al. 1999, 2000, 2001). The copper deficiency is associated with a decreased bone strength and deterioration of bone quality leading to osteoporotic defects (Medeiros et al. 1997).

In this study, we present the synthesis of some analogues of sialorphin to investigate in what way some structural modification may have an impact on coordination abilities of the peptides. We present the interaction of new pentapeptides with Cu(II) ions over a wide pH range. To determine the coordination model of ligands, a series of measurements using the spectroscopic (UV–Vis, CD) and potentiometric techniques has been performed.

## Experimental

### Peptide Synthesis and Purification

All of the peptides were synthesized on a solid-phase method using standard Fmoc procedures on a 2-chlorotrityl chloride resin (loading 0.3–0.9 mmol/g, 1% DVB, 200–400 mesh, Orpegen Peptide Chemicals GmbH, Heideberg, Germany). *N*-α-protected amino acids, and reagents used for the solid-phase synthesis were obtained from Iris Biotech GmbH (Marktredwitz, Germany). Peptides chains were elongated in the consecutive cycles of deprotection and coupling. Two deprotection steps using 20% piperidine in dimethylformamide (DMF) (5 and 15 min) were performed. The coupling reactions were carried out with a threefold molar excess of the protected amino acid (Fmoc-AA) dissolved in DMF/dichloromethane (DCM) using *N,N′*-diisopropylcarbodiimide (DIC) and *N*-hydroxybenzotriazole (HOBt) for 2 h (Fmoc-AA:HOBt:DIC, 1:1:1). The completeness of each coupling step was monitored by the chloranil test. The peptides were cleaved from the resin and the protecting groups were removed in one step using a mixture of TFA/triisopropylsilane/H_2_O (95:2.5:2.5, v/v/v). The cleaved peptides were precipitated with cold diethyl ether and lyophilized.

The peptides were purified using the RP-HPLC on a Kromasil C8 column (8 × 250 mm, 100 Å pore size, 5 µm particle size) with linear gradient 2–40% of acetonitrile in 0.1% TFA for 30 min with a flow rate of 10 mL/min. The purity of the peptides was checked on a Beckman HPLC controlled by Lp-Chrom system. Fractions containing the pure peptides (>98%) were pooled and lyophilized. The mass spectrometry analysis were carried out on a MALDI-TOF MS. Physicochemical characteristics of all synthesized peptides are presented in Table 1.

**Table 1:** Physicochemical characteristics of investigated peptides

Peptide	Sequence	Formula	HPLC R_t_ (min)	[M]^+^_calc__._	[M + H]^+^_found_
Opiorphin	QRFSR	C_29_H_48_N_12_O_8_	6.7^a^	692.4	693.3
Sialorphin	QHNPR	C_26_H_42_N_12_O_8_	5.5^a^	650.3	651.2
P1	QRFPR	C_31_H_50_N_12_O_7_	7.87^b^	702.4	703.1
P2	AHNPR	C_24_H_39_N_11_O_7_	5.37^b^	593.29	594.8
P3	QANPR	C_23_H_40_N_10_O_8_	5.21^b^	584.29	585.0

^a^Linear gradient from 2 to 40% of [B] in [A] for 15 min, flow rate of 1.5 mL/min, Kromasil C8 column (5 μm, 4.6 × 250 mm), where [A] is 0.1% TFA in H_2_O and [B] is 0.1% TFA in acetonitrile

^b^Linear gradient from 2 to 60% of [B] in [A] for 15 min, flow rate of 1.5 mL/min, Kromasil C8 column (5 μm, 4.6 × 250 mm), where [A] is 0.1% TFA in H_2_O and [B] is 0.1% TFA in acetonitrile

### Potentiometry Measurements

The potentiometry titrations were conducted on a MOLSPIN pH-meter system, using a Mettler Toledo InLab^®^Micro combined electrode. The pH-meter system was calibrated by triple titration of hydrogen ion (HCl) before each measurement. The potentiometric experiments were performed in argon atmosphere over the pH range 3.0–11.5 at 298 K. The ligands were dissolved in HCl/KCl where pH = 3.0 and I = 0.1 M (KCl), the ligands concentration were 1 × 10^−3^ M. The accurate concentration of ligands solutions were determined by Gran method. The samples of each titration were prepared by adding equimolar amounts of CuCl_2_ solutions. Titration volume were 1–1.5 ml and alkali (c(KOH) ~0.1 M) was added from a 0.250 ml micrometer syringe. The stability constants for the proton (β_i_^(H)^) and Cu(II) complexes (β_(CupHqLr)_) were calculated by HYPERQUAD 2008 (written by Peter Gans, Protonic Software) and SUPERQUAD computer programs (Gans et al. 1985, 1996). The protonation constants of ligands is determined by: β_i_ = [H_i_L]/[H^+^]^i^[L] and the equilibrium of complex stability: β_pqr_ = [M_p_H_q_L_r_]/[M]^p^[H]^q^[L]^r^. Standard deviations were computed by these both programs and refer to random errors only.

### Spectroscopy Measurements (UV–Vis, CD)

All spectroscopic measurements were carried out at 298 K. The solutions of samples were prepared in similar way like samples used in pH-metric titration. The pH of the samples were determined by adding small quantity of concentrated solutions of KOH. The UV–Vis absorption spectra of complexes were recorded on Varian Carry 50 Bio spectrophotometer. All spectra were collected in quartz cells with 1 cm path length and spectral range was 230–800 nm. For each spectra were calculated the molar extinction coefficients [ε (M^−1^ cm^−1^)] at a wavelength of maximum absorption. The circular dichroism (CD) spectra were recorded on a magnetic circular spectrometer JASCO J-1500. The measurements were conducted in 0.5 cm cuvettes in the range 240–800 nm with 0.1 nm resolution. The measurements were carried out in an inert gas atmosphere (N_2_). The CD spectral data were recalculated to the molar ellipticity [ε (M^−1^ cm^−1^)].

## Results and Discussion

**Scheme 1:**
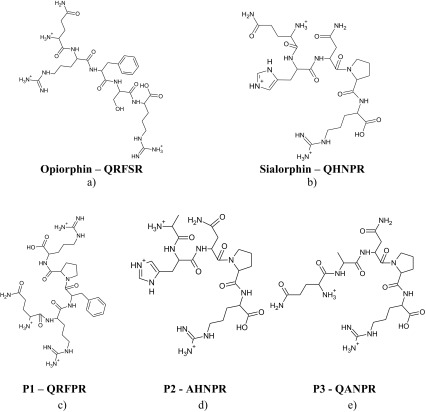
The full protonated structure of: **a** opiorphin, **b** sialorphin, and their analogues: **c P1**–QRFPR, **d P2**–AHNPR, **e P3**–QANPR

The presented manuscript shows the binding abilities of three novel analogues of saliva pentapeptides: opiorphin and sialorphin (Scheme 1a, b). The characteristic feature of these ligands is the presence the glutamate amino acid residue in the position 1 of the peptide chain. Moreover sialorphin has His and Pro moieties located at position 2 and 4 of the peptide chain (Scheme 1b). These two residues significantly influence the process of the metal ion binding (Kozłowski et al. 1999; Pettit et al. 1985; Sovago and Osz 2006; Sovago et al. 2016).

The modification of the opiorphin has been performed by the insertion of proline residue instead of the serine located at position 4 (**P1**, Scheme 1c). Next two analogues have been created by the insertion of alanine residue instead of Gln-1 (**P2**, Scheme 1d) or His-2 (**P3**, Scheme 1e) in the sialorphin sequence. The detailed analysis of the results obtained for these analogues in comparison to the results for the parent peptide allows to characterize the influence of the specific amino acid resides as Gln, His, Pro or Asn on the coordination abilities of two selected saliva peptides.

The first step of the presented studies is the analysis of the results obtained for the analogue of opiorphin (**P1**). The acid-base properties as well as the stabilities constants of formed complexes and the spectral abilities are presented in Table 2.

**Table 2:** Protonation constants of P1–QRFPR and stability constants with the spectroscopic parameters for its Cu(II) complexes at T = 293 K, I = 0.1 M (KCl)

P1		
Species	log*β*	p*K*
HL	11.34 ± 0.04	11.34 (Arg)
H_2_L	18.42 ± 0.05	7.08 (-NH_2_)
H_3_L	21.53 ± 0.05	3.11 (-COO^−^)

Cu(II) complexes						
Species/binding model	log*β*	p*K*	UV–Vis		CD	
			λ(nm)	ε(M^−1^ cm^−1^)	λ(nm)	ε(M^−1^ cm^−1^)
CuHL {NH_2_, 3H_2_O}	16.42 ± 0.08		680	47	315	0.25
CuL {NH_2_, N^−^_am_, 2H_2_O}	10.71 ± 0.04	7.33	640	89	566	−0.41
					315	0.89
					268	−1.22
CuH_−1_L {NH_2_, 2N^−^_am_, H_2_O}	3.87 ± 0.05	9.39	573	138	569	−0.85
					315	1.02
					275	−1.03
CuH_−2_L {NH_2_, 2N^−^_am_, OH^−^}	−5.64 ± 0.07	11.14	573	134	562	−0.85
					315	0.97
					270	−1.75
CuH_− 3_L {NH_2_, 2N^−^_am_, OH^−^}	−17.03 ± 0.11		579	112	545	−0.80
					315	0.63
					270	−3.43

**Fig. 1:**
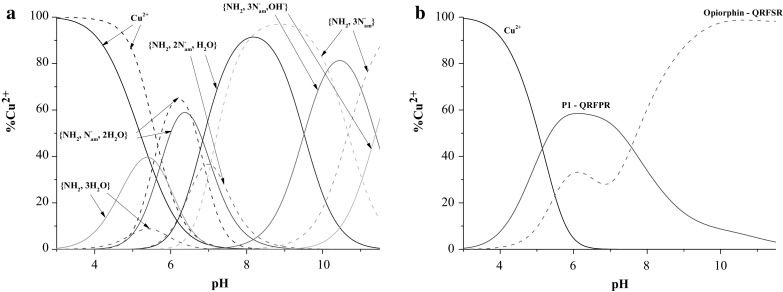
**a** The species distribution curve of formed Cu(II) complexes in dependence on pH for P1 (*solid line*) and opiorphin (*dashed line*). **b** The diagram for competitive binding of Cu(II) ions by P1 (*solid line*) and opiorphin (*dashed line*) ligands. The opiorphin:Cu(II):P1 molar ratio is 1:1:1, C = 1 × 10^−3^M

The ligand **P1** forms five types of complexes as its parent peptide (Fig. 1a; Table 1). The metal ion binding by **P1** starts around pH 4 by the formation of the CuHL species, which achieves its highest concentration at pH 5.5 (Fig. 1a). The value of log*β* ^*^ = 5.08, were log*β* ^*^ = log*β*_*CuHL*_ − log*β*_*HL*_, is comparable to stabilities calculated for the complexes with the N terminal amino group involved in Cu(II) binding (Prenesti et al. 1999). Moreover, the λ_max_ for d-d transition at 680 nm confirms the involvement only one nitrogen donor in metal ion binding (Prenesti et al. 2006).

**Scheme 2:**
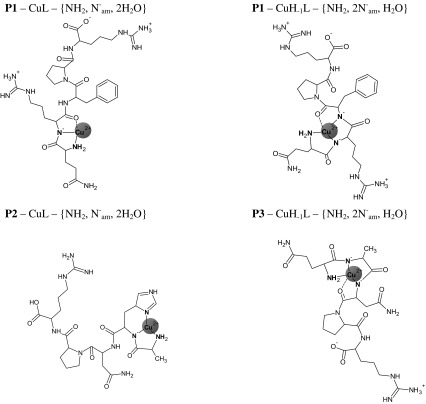
The schematic structures of the Cu(II) complexes existing in the salivary range of pH (6.5–7.5) in the system with ligands P1, P2 and P3. The water molecules were omitted to more clarity

Afterwards, with the increase of pH, the involvement of the first and the second amide takes place and formation of the species with the {NH_2_, N^−^_am_, 2×H_2_O}, {NH_2_, 2×N^−^_am_, H_2_O} (Scheme 2) donor atoms in the plane is observed, consecutively. The location of the λ_max_ for d–d transitions at ^CuL^640 nm and ^CuH−1L^573 nm are in good agreement with the theoretical λ_max_ = 650, 576 nm calculated for proposed donor sets (Billo 1974; Prenesti et al. 1999). Moreover, the involvement of the N terminal amino as well as amide donors in Cu(II) coordination is supported by presence of one negative and one positive CT bands at 270 and 320 nm (Table 2), consecutively. Up to pH 7, the process of Cu(II) binding by the unmodified and modified opiorphin is carried out in the same manner. Nevertheless, the insertion of proline in position 4 promotes the formation of the species with {NH_2_, 2xN^−^_am_, H_2_O} coordination. Above last to complexes appear in the investigated system: CuH_−2_L and CuH_−3_L, and this process does not influences significantly the spectral abilities of the system (Table 2).

As it was mentioned above in the case of opiorphin the formation next two species is observed at significantly lower range of pH. Moreover the unmodified opiorphin, involves third amide donor in Cu(II) binding and forms the species with the {NH_2_, 3xN^−^_am_} binding manner (Kotynia et al. 2010). This ability is associated with the absence of the Pro-4 in the peptide backbone. The proline is the amino acid residue which strongly influences the binding ability of the peptide. When proline is present in the peptide chain starting from the position 2, it effectively prevents binding of the subsequent amide donors (Bataille et al. 1984; Formicka-Kozlowska et al. 1981; Kozłowski et al. 1999). Owing to this fact and any significant changes in the spectral abilities of the system above pH 9,5, the formation of the CuH_−2_L and CuH_−3_L complexes may be assigned to proton dissociation form the metal bound water molecule(Holm et al. 1996; Jakab et al. 2008; Kamysz et al. 2013) and guanidyl group of the side chain of arginine moiety (Fitch et al. 2015; Holm et al. 1996; Matera et al. 2008).

As it was mentioned, the insertion of the proline in the fourth position of the peptide backbone effectively prevents the involvement of the subsequent amide donor, however due to this fact, the system with the ligand **P1** is more specific and selective in comparison to the unmodified opiorphin.

The investigations of the coordination abilities of two new analogues of sialorphin: **P2** and **P3** (Scheme 1 d, e) were the next step of our studies. The modification in the case of the first sialorphin analogue (**P2**) consisted in the replacement of Gln-1 by alanine moiety and it does not influences the binding abilities of **P2**. It forms the same type of dominant complexes as unmodified ligand (Kamysz et al. 2013). The species CuL dominating at physiological range of pH is the characteristic complex for His-2 peptides (Kozłowski et al. 1999) with three nitrogen donors from: N terminal amino group, first amide and imidazole ring for His-2 moiety (Scheme 2). The presence of these donors in the coordination sphere of Cu(II) ion is supported by the CT transitions at: 340 and 263 nm (Table 3) in CD spectrum. Moreover the location of the d–d band at 600 nm (Table 3) is almost the same as theoretical λ_max_ = 599 nm, calculated for the –NH_2_, N^−^_am_, N_Im_, H_2_O chromophores, what supports the postulated coordination mode. The appearance of the final species significantly influences the spectral abilities as the red shift of the λ_max d–d_ 600 nm → 528 nm what suggests four nitrogen donors in the square plane symmetry (Casolaro et al. 1999; Marchewka et al. 2002). The formation such complex is possible when: the N terminal amino group, two amides and amide form the side chain if Asn-3 are bound to Cu(II). The same type of the species was found for the sialorphin (Kamysz et al. 2013).

**Table 3:** Protonation constants of peptides P2–AHNPR and P3–QANPR and stability constants with the spectroscopic parameters for Cu(II) complexes at T = 298 K, I = 0.1 M (KCl)

P2		
Species	log*β*	p*K*
HL	∼11.91 ± 0.09	∼11.91 (Arg)
H_2_L	19.83 ± 0.09	7.92 (–NH_2_)
H_3_L	26.01 ± 0.10	6.19 (N_Im_)
H_4_L	29.11 ± 0.10	3.10 (–COO^−^)

Cu(II) complexes						
Species/binding model	log*β*	p*K*	UV–Vis		CD	
			λ (nm)	ε (M^−1^ cm^−1^)	λ (nm)	ε (M^−1^ cm^−1^)
CuH_2_L {NH_2_, 3H_2_O}	25.22 ± 0.04		–	–	–	–
CuL {NH_2_, N^−^_am_, N_im_, H_2_O}	17.89 ± 0.01	7.33	600	68	607	0.37
					511	−0.07
					340	0.26
					263	2.35
CuH_−1_L {NH_2_, 2N^−^_am_, N_im_}	8.50 ± 0.02	9.39	575	66	585	0.33
					480	−0.07
					320	0.28
					260	2.44
CuH_−2_L {NH_2_, 2N^−^_am_, NH^−^_Asn3_}	−2.64 ± 0.03	11.14	528	85	618	0.10
					504	0.13
					309	0.70
					274	−1.16

P3		
Species	log*β*	p*K*
HL	∼11.19 ± 0.04	∼11.19 (Arg)
H_2_L	18.62 ± 0.05	7.43 (–NH_2_)
H_3_L	21.98 ± 0.06	3.36 (–COO^−^)

Cu(II) complexes						
Species/binding model	log*β*	p*K*	UV–Vis		CD	
			λ (nm)	ε (M^−1^ cm^−1^)	λ (nm)	ε (M^−1^ cm^−1^)
CuHL {NH_2_, 3H_2_O}	16.74 ± 0.03		678	15	–	–
CuH_−1_L {NH_2_, 2N^−^_am_, H_2_O}	5.78 ± 0.02	10.96	550	98	590	−0.37
					500	0.19
					311	0.65
					276	−1.43
					243	1.90
CuH_−2_L {NH_2_, 2N^−^_am_, OH^−^}	−2.96 ± 0.03	8.74	544	95	580	−0.37
					493	0.19
					310	0.61
					274	−1.57
					243	1.50
CuH_−3_L {NH_2_, 2N^−^_am_, NH^−^_Asn3_}	−12.83 ± 0.03	9.87	520	81	562	−0.50
					493	0.34
					305	0.80
					268	−2.10

**Fig. 2:**
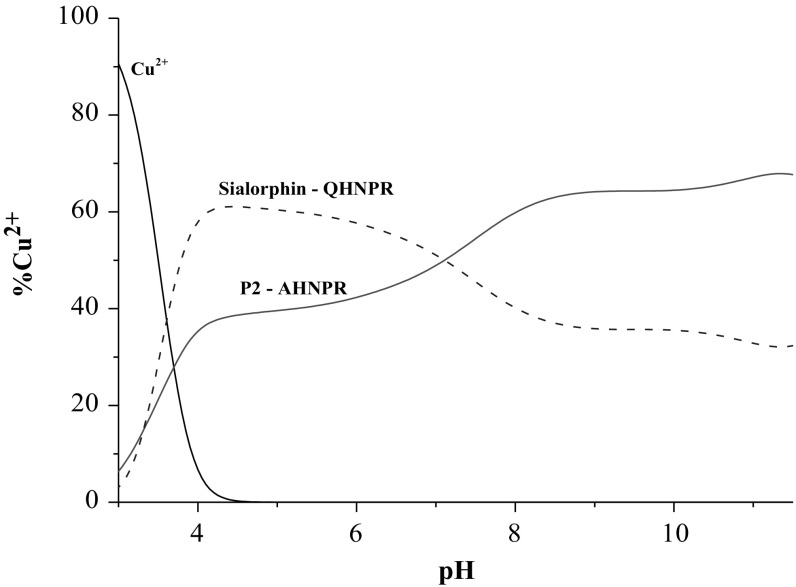
The diagram for competitive binding of Cu(II) ions by **P2** and sialorphin. The sialorphin:Cu(II):P1 molar ratio is 1:1:1, C_l_ = 1 × 10^−3^M

As it was presented above, the modification does not influences significantly the binding abilities of the peptide however the efficiency in Cu(II) binding changes (Fig. 2). Below pH 8, the **P2** is around 20% less effective in Cu(II) binding however its efficiency significantly increases above this pH. It is probably caused by the steric changes induced less developed side chain of alanine than glutamine.

The interesting results have been obtained for the third from investigated peptide **P3** (Scheme 1e) consisting alanine in position 2 instead of His moiety. Moreover, due to the presence of Pro-4 residue in the peptide chain it can be also assigned as the analogue of **P1**. The comparison of the coordination abilities between **P3** and **P1** and sialorphin, shows that **P3** has higher similarity in copper coordination as **P1** than sialorphin.

**Fig. 3:**
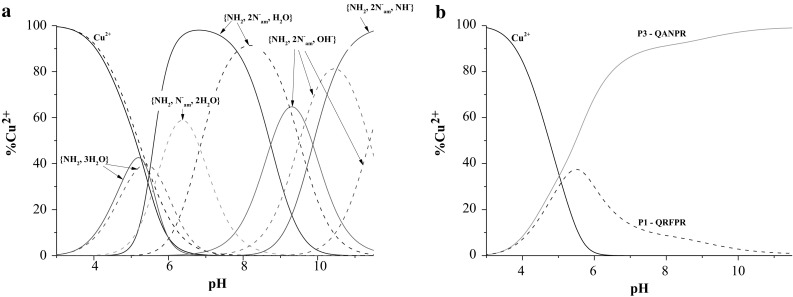
**a** The species distribution curve of formed Cu(II) complexes in dependence on pH for P3 (*solid line*) and P1 (*dashed line*). **b** The diagram for competitive binding of Cu(II) ions by P3 (*solid line*) and P1 (*dashed line*) ligands. The P3:Cu(II):P1 molar ratio is 1:1:1, C = 1 × 10^−3^M

**P3** starts Cu(II) binding by formation of the CuHL species with metal ion anchored by the N terminal amino group (Fig. 3a), as it was found in a case of **P1**. Then with increase of pH simultaneous dissociation of two protons take place and the CuH_−1_L appears in the system. This process is correlated with the involvement of the next two nitrogen donors binding to copper ion, what is confirmed by the spectroscopic results obtained for the CuH_−1_L species (Table 3, Scheme 2).

The presence of CT transitions at 311, 276 and 243 nm (Table 3) in CD spectrum confirm the involvement of the amido and amino donors in copper (II) binding. Moreover, the location of d–d supports involvement of three nitrogen donors in copper binding. Owing to these facts, the discussed complex can be characterized by the {NH_2_, 2×N^−^_am_, H_2_O} binding mode and it dominates in salivary range of pH as in a case of **P1** (Scheme 2).

With increase of pH last two species exist in the system. First of them is the CuH_−2_L, which achieves its highest concentration at pH 9.5 (Fig. 3a). The spectroscopic parameters of the system at pH 9.5 do not change significantly (Table 3) what may support the proton dissociation form the metal bound water molecule (Holm et al. 1996; Jakab et al. 2008; Kamysz et al. 2013). The spectral abilities of the investigated system dramatically change above pH 10, in which the dominant is the CuH_−3_L species. The most of all the shift of λ_max_ to 520 nm (Table 3) strongly support the involvement of the fourth nitrogen in copper binding and formation of the complex with square planar symmetry.

**Scheme 3:**
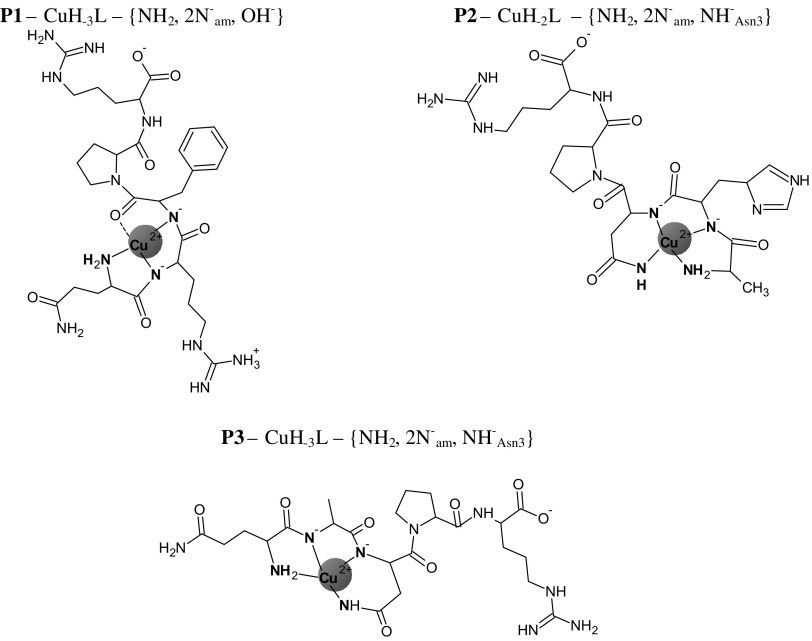
The schematic structures of the final Cu(II) complexes existing in basic pH in the system with ligands P1, P2 and P3

Based on the peptide structure and the previous results for sialorphin (Kamysz et al. 2013), there is only one possibility to involve fourth nitrogen in Cu(II) coordination: it is the amide nitrogen from the side chain of Asn-3 residue (Scheme 3). The same process was also observed in a case of **P2** (Scheme 3) as well as unmodified sialorphin (Kamysz et al. 2013).

Despite the fact, that both peptides form the same type of dominant complexes, except the last one, they show different efficiency in metal binding (Fig. 3b). Starting already from pH 5 the **P3** is getting to be significantly more effective in copper binding as the **P2**. This difference may be caused by the fact that the **P3** has alanine residue at position 2 of the peptide backbone. Alanine is the amino acid with simpler side chain in comparison to arginine, which is present in the second position of the **P1** peptide chain and the simultaneously involvement of two amide donors and formation of the more stable species with the {NH_2_, 2N^−^_am_} is observed already at pH 5 in contrast to **P1** (Fig. 3a).

## Conclusions

In the presented manuscript we have characterized coordination abilities of new analogues of the saliva peptides (opiorphin and sialorhin) toward Cu(II) ions. The detailed analysis of the results obtained for these analogues in comparison to the results for the parent peptides allowed to characterize the influence of the specific amino acid resides as Gln, His, Pro or Asn on the coordination abilities of two selected saliva peptides as well as the efficiency in metal binding. The knowledge on coordination abilities of salivary peptides and their analogues by copper ions is crucial for a rational drug design of new therapeutic agents that could be involved in the regulation of the level of copper ions.
